# The inhibitor miR-21 regulates macrophage polarization in an experimental model of chronic obstructive pulmonary disease

**DOI:** 10.18332/tid/140095

**Published:** 2021-09-02

**Authors:** JunJuan Lu, LiHua Xie, ShengHua Sun

**Affiliations:** 1 Department of Respiratory Medicine, The Third XiangYa Hospital of Central South University, Changsha, People’s Republic of China

**Keywords:** macrophage polarization, chronic obstructive pulmonary disease, miR-21, cigarette smoke extract

## Abstract

**INTRODUCTION:**

In chronic obstructive pulmonary disease (COPD), macrophages play an indispensable role. In the lung tissues of COPD patients and smokers, macrophages can be observed to polarize towards M2 phenotype. The molecular mechanism of this process is unclear, and it has not been fully elucidated in COPD.

**METHODS:**

We bought laboratory animals [C57BL/6 and miR-21^–/–^ C57BL/6(F1)] from the Jackson Laboratory. The model of COPD mice was established by cigarette smoke (CS) exposure combined with intraperitoneal injection of cigarette smoke extract (CSE). RT-PCR detected the expression levels of inflammatory factors and markers associated with M1 and M2 macrophages. The ratio of M2 macrophages to M1 macrophages was detected by immunohistochemical staining.

**RESULTS:**

The level of miR-21 was increased in RAW264.7 cells intervened by CSE and in lung tissue and bone marrow-derived macrophages (BMDMs) from COPD mice. CSE can gradually over time increase the level of miR-21. The proportion of M2 macrophages to M1 macrophages had a positive correlation with miR-21. Knockdowning miR-21 can reduce lung tissue damage. CSE also increased the levels of related inflammatory factors and markers associated with M2 macrophages, and an miR-21 inhibitor can reverse this conversion.

**CONCLUSIONS:**

We confirmed that CSE can lead to macrophage transformation to the M2 phenotype and an increase in the expression level of miR-21. Knockdown of the miR-21 gene could inhibit the transformation of macrophages to the M2 phenotype in COPD.

## INTRODUCTION

COPD is one of the diseases with high economic burden and mortality in the world. It is estimated that more than 300 million people were affected by COPD in 2020 in the world, and 4.7 million of the 68 million deaths are caused by COPD^1-3^. The number of macrophages is increasing in the small airways of COPD patients^4^, and there is a close relationship between the number of macrophages and the severity of COPD^4,5^. Multiple studies have shown that the number of macrophages in the lung tissue of COPD patients is increased; in addition, there is a correlation between disease progression and the number of macrophages^5,6^. Alveolar macrophages (AM) play an indispensable role in the occurrence and development of COPD, and, at the same time, it can affect the structure of lung tissue through the initiation and degeneration of inflammation^7^.

Mutative exogenous stimuli and pathological processes can change the phenotypes and functional characteristics of alveolar macrophages^8^. Since a large number of M1-type macrophage-related cytokines, such as TNF-α and IL-8, are found in bronchoalveolar lavage fluid (BAL), pulmonary macrophages from patients with COPD have been recommended to display M1 phenotypes^9^. In addition, TNF-α has been shown to be closely related to cigarette smoking-induced emphysema in mice^10^. Research has shown that CS can induce macrophages to reprogram to the M2 phenotype in patients with COPD^11^. In vitro, using different concentrations of CSE to interfere with macrophages, the expression of M2 macrophagerelated markers (CD163, CD204, and CD206)^12-14^ and related factors (IL-10, TGF-β1, TGF-β2) was up-regulated^15,16^. Similarly, our previous studies showed that the proportion of M2 macrophages to M1 macrophages in BAL and lung tissue in a COPD mouse model were both significantly increased^17,18^. A number of etiology factors may contribute to changes in macrophage phenotypes including gram-negative anaerobe actinomycetes (AA), smoking and porphyromonas gingival (PG), and smoking is the most important factor^19^. However, the regulatory mechanism of macrophage polarization in COPD is unclear.

MicroRNAs (miRNAs) are small endogenous non-coding RNAs which bind in a sequence-specific manner to incomplete complementary sites in target messenger RNA (mRNA), thereby directly inhibiting protein translation or transcriptome degradation. In this way, miRNAs can simultaneously interact with hundreds of genes and regulate various developmental and physiological processes, including cell proliferation, differentiation, apoptosis, and innate and adaptive immune responses^20^. The research shows that miRNAs influence macrophage activation and polarization^21^. Our previous study confirmed that miR-21 was increased in lungs from a COPD mouse model and the peripheral blood of COPD patients, and its expression level correlated with lung function^22^. However, whether miR-21 is involved in COPD development by targeting macrophage polarization is unknown.

Therefore, in this study, we used CS exposure combined with intraperitoneal injection of CSE to construct miR-21^–/–^ COPD animal models, to observe the effect of CSE on miR-21 and investigate the possible role of miR-21 in COPD-associated macrophage polarization.

## METHODS

### Animals, preparation of CSE and animal model

C57BL/6 and miR-21^–/–^ C57BL/6(F1) mice were randomly divided into a control group and a COPD model group, all of which were purchased from the Jackson Laboratory. All animals were kept in clean rooms with a temperature of 23–25°C, a humidity of 50–60% and a 12-hour light/dark cycle. The study followed the guidelines for animal and human research^23^. The preparation of CSE followed previous procedure^24^. The COPD mice model was established by CS exposure combined with intraperitoneal injection of CSE. The whole experiment lasted 28 days. The modeling box was made as previously described^25^. First, five cigarettes were lit simultaneously for 15 minutes. Second, the box is opened and the animals allowed to rest for five minutes. Then, the first step is repeat. The entire process is called CS exposure cycle, with exposure twice a day during the entire experimental cycle, except for the 1st, the 12th, and 23rd day; the control group was kept in the experimental animal center of the Third Xiangya Hospital of Central South University. The intraperitoneal injection of CSE was carried out according to the previous method. On days 1, 12, and 23, the control animals were intraperitoneally injected with 0.3 mL/20 g PBS, while the model animals were intraperitoneally injected with 0.3 mL/20 g CSEPBS. On day 29, the lung function of the mice was measured, and lung tissue and BMDMs were collected. This research was approved by the Institutional Review Board of Central-South University and followed the guidelines for animal and human research^23^.

### Cell culture and production of BMDMs

RAW264.7 and L929 cell lines were purchased from the American Type Culture Collection and were cultured using DMEM (Life Technologies, Carlsbad, CA, USA) containing 10% (v/v) heat-inactivated FBS. According to our previous experiments, CSE interferes with RAW264.7 cells at a concentration of 5%. BMDMs are established as previously described^26^.

### Histomorphology of lung tissue

The left lower lung was lavaged with 4% paraformaldehyde, and then fixed with 4% paraformaldehyde overnight^27^. The lung tissue was embedded in paraffin (Sigma, MO, USA) , cut it into 4 μm sections after embedding, and stained with hematoxylin and eosin (H & E) (Sigma). The three indicators of mean linear intercept (MLI), mean alveolar interval thickness (MAST) and destruction index (DI) were used to quantify the pathological severity of emphysema. The measurement methods have been previously described^25^.

### Immunohistochemical staining

The dewaxing and descaling sections were incubated with 1% H_2_O_2_ at room temperature for 30 minutes to eliminate endogenous peroxidase activity, and then anti-CD68, anti-CD206 and anti-CD86 antibodies (ProInTech, Chicago, IL, USA) were added and incubated overnight at 4°C. The sections were washed thoroughly on the second day and incubated with the secondary antibody for 1 hour at room temperature. The secondary antibody needs to be coupled with appropriate horseradish peroxidase. The unreacted secondary antibody was removed and the section was incubated with 3,39-diaminobiphenyl-4HCl (DAB; Sigma, MO, USA) -H_2_O_2_ solution to observe the immunolabeling, and then HE was used to counterstain the section using a fixed glass cover. The presence of CD68, CD206 and CD86 can be indicated by varying degrees of tan or brown particles or flake deposits. Four or five CD68, CD206 and CD86 positive images were randomly selected to calculate the integrated photometric value (IOD) and average optical density (AOD). Image-Pro Plus version 6.0 (Media Cybernetics, Inc., Rockville, MD, USA) software was used for high magnification of each slice to visualize high-definition color pathological images and analyze the results.

### Transient miRNA transfections

The miRNA-21 inhibitor was purchased from Qiagen, and the all-star negative mimic (Qiagen) was used as a control. According to the transfection reagent instructions, the cells were transiently transfected with Hiperfect Transfection Reagent (Qiagen) at a final concentration of 50 nM. Red siGLO oligos (Thermo Fisher Scientific, Waltham, MA, USA) was used to confirm the transfection efficiency of the cells. The transfection efficiency was greater than 90% and the transfection was considered successful^28^.

### Quantitative real-time PCR

According to the instructions, TRIzol reagent (Takara, Dalian, Liaoning, China) is used to isolate total RNA, and SYBR Green PCR Master Mix (Bio-Rad, CA, USA) used to detect mRNA levels. The mRNA expression was detected using a two-step quantitative real-time polymerase chain reaction (Applied Biosystems, Carlsbad, CA, USA). As endogenous control β-actin was used, and the DD cycle threshold method was used to determine the relevant expression level. Table 1 identifies the primer sequences.

**Table 1:** Primer sequences of the genes investigated in RT-PCR analysis

*Gene*	*Primer sequences*
Β-Actin	F 5'-AACGGCTCCGGCATGTGCAA-3'
	R 5'-CTTCTGACCCATGCCCACCA-3'
TNF-a	F 5'-CAGCCTCTTCTCCTTCCTGAT-3'
	R 5'-GCCAGAGGGCTGATTAGAGA-3'
IL-10	F5'-ACCAAGACCCAGACATCA-3'
	R 5'-TTCACAGGGAAGAAATCG-3'
IL-6	F 5'-GGAACTCTACCAGAAATATAGC-3'
	R 5'-CCTGTATTCCGTCTCCTTG-3'
CD206	F 5'-GTGGAGTGATGGAACCCCAG-3'
	R 5'-CTGTCCGCCCAGTATCCATC-3'
Ym1	F 5'-GAAGGAGCCACTGAGGTCTG-3'
	R 5'-TGTTGTCCTTGAGCCACTGA-3'
Fizz1	F 5'-CGTGGAGAATAAGGTCAAGGA-3'
	R 5'-CAGTAGCAGTCATCCCAGCA-3'
ARG1	F 5'-GCATATCTGCCAAAGACATCG-3'
	R 5'-CCATCACCTTGCCAATCCC-3'
iNOS	F 5'-AGGGAATCTTGGAGCGAGTT-3'
	R 5'- GCAGCCTCTTGTCTTTGACC-3'

### Statistical analysis

The results were described using mean ± standard deviation, and GraphPad Prism Software 6.0 (GraphPad Software, La Jolla, CA, USA) was used for data analysis. One-way analysis of variance was used for statistical evaluation, and the correlation between groups was analyzed using the Pearson correlation coefficient. A p<0.05 was considered statistically significant.

## RESULTS

The expression of miR-21 is up-regulated in RAW264.7 cells intervened by CSE and lung tissue and BMDMs from COPD mouse model.

**Figure 1:**
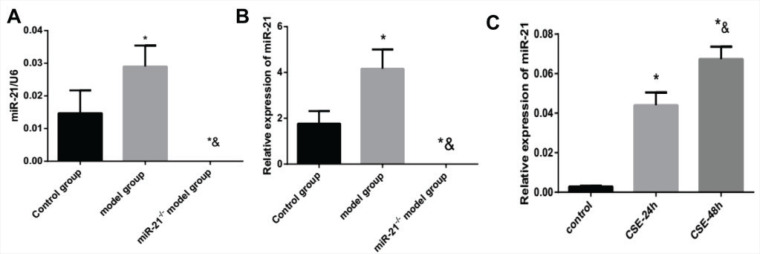
The expression of miR-21 is up-regulated by cigarette smoke extracts. A: the expression level of miR-21 in lung tissue. Control group: normal control group. Model group: C57BL/6 mice were intervened by CS exposure combined with CSE intraperitoneal injection. The miR-21–/– model group: miR-21–/– C57BL/6 mice were intervened by CS exposure combined with CSE intraperitoneal injection. *p<0.05, versus the control group; *&p<0.05, versus COPD model group. B: the expression level of miR-21 in BMDMs.*p<0.05, versus the control group; *&p<0.05, versus COPD mouse model. C: the expression level of miR-21 in RAW264.7 cells treated with CSE. *p<0.05, versus the control group. *&p<0.05, versus the CSE-24h group.

Our experiment detected the expression of miR-21 in RAW264.7 cells intervened by CSE and lung tissue and BMDMs from COPD mouse model and miR-21^-/-^ mice using RT-PCR. The RAW264.7 cells were intervened by 5% CSE for 24 h and 48 h *in vitro*. Compared with the control group, the expression of miR-21 in lung tissues and BMDMs of COPD mice was significantly increased (p<0.05, Figures 1A and 1B). Moreover, CSE stimulation increased the expression of miR-21, which gradually rose over time (p<0.05, Figure 1C).

### The change in M2/M1 macrophages and its correlation with miR-21

**Figure 2:**
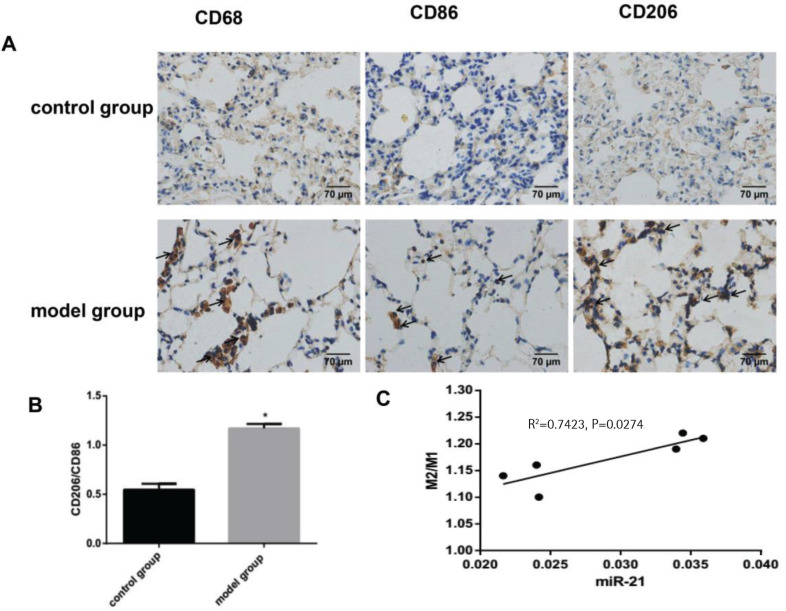
The change in M2/M1 macrophages and its correlation with miR-21. Control group: normal control group. Model group: C57BL/6 mice were intervened by CS exposure combined with CSE intraperitoneal injection. A: the protein expression levels of CD68, CD86 and CD206 as well as immunohistochemical staining for CD206 expression in lung tissue from COPD mouse model showing increased deposition of brown granules in cell membranes (black arrows, 400×magnification). B: ratio of M2/M1 macrophages. *p<0.05, versus the control group. C: ratio of M2 /M1 macrophages and its correlation with miR-21.

In this experiment, immunohistochemical staining showed a rising proportion of M2 macrophages to M1 macrophages, which indicated the macrophages in the lung tissue of COPD mice had a tendency to polarize towards M2 macrophages (p<0.05, Figures 2A and 2B). Furthermore, the proportion of M2 macrophages to M1 macrophages was positively correlated with the expression of miR-21 (p<0.05, Figure 2C). In our COPD mice induced by the combination of CS exposure and intraperitoneal injection of CSE, macrophages were more likely to have the M2 phenotype.

### The pathological morphological changes in lung tissue after knocking out miR-21

**Figure 3:**
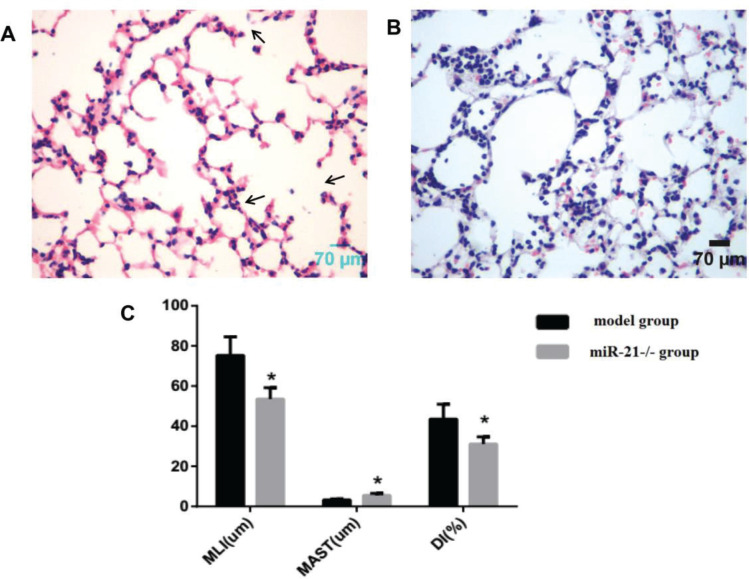
The pathological morphological changes of lung tissue after knocking out miR-21 while using the same method to build the COPD model. Model group: C57BL/6 mice were intervened by CS exposure combined with CSE intraperitoneal injection. The miR-21–/– group: miR-21–/– C57BL/6 mice were intervened by CS exposure combined with CSE intraperitoneal injection. A: HE staining of lung tissue from model mice (400×magnification) showing an enlarged alveolar space, a thinner alveolar septum and a destroyed alveolar wall (black arrows). B: HE staining of lung tissue from miR-21-/- mice (400×magnification) showing increased alveolar septum thickness, reduced alveolar size and destruction of the alveolar wall. C: comparison of lung histomorphology in the model group and the miR-21-/- group. MLI (μm): mean linear intercept. MAST (μm): mean alveolar septal thickness. DI (%): destruction index. *p<0.05, versus the model group.

Using the same way to build the COPD mouse model, the alveolar septum thickness was increased compared to wild-type mice, and the alveolar size and destruction of the alveolar wall were reduced in miR-21 knockout mice (Figures 3A and 3B). MLI and DI were reduced, and MAST was increased in miR-21 knockout mice (p<0.05, Figure 3C).

### The effect of CSE and miR-21 on macrophage polarization in BMDMs and RAW264.7 cells

**Figure 4:**
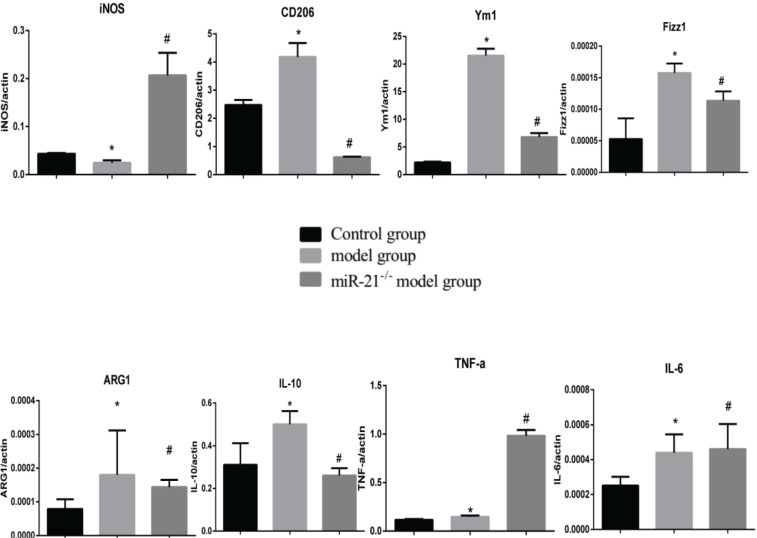
RNA expression levels of M1/M2 macrophages-related cytokines and markers in BMDMs from the control, COPD and miR-21–/– COPD groups. Control group: normal control group. Model group: C57BL/6 mice were intervened by CS exposure combined with CSE intraperitoneal injection. The miR-21–/– model group: miR-21–/– C57BL/6 mice were intervened by CS exposure combined with CSE intraperitoneal injection. *p<0.05, versus the control group. #p<0.05, versus the model group.

**Figure 5:**
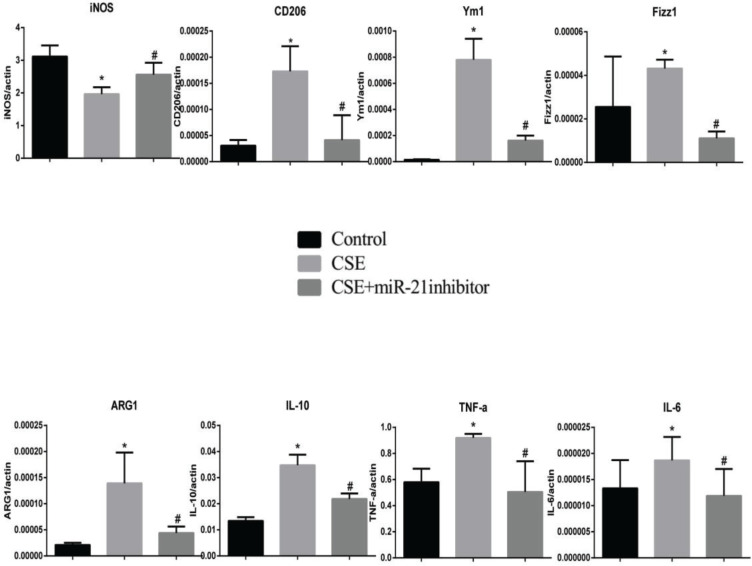
RNA expression levels of M1/M2 macrophages-related cytokines and markers in RAW264.7 cells from the control, cigarette smoke extract (CSE)-treated and miR-21 inhibitor-treated groups. CSE: 5% CSE interfering with RAW264.7 cells. CSE+ miR-21 inhibitor: miR-21 inhibitor interfering with CSE-treated RAW264.7 cells. *p<0.05, versus the control group. #p<0.05, versus the CSE group.

To clarify the effects of CSE and miR-21 on the polarization of macrophages, we used RT-PCR to detect the expression levels of inflammatory factors and markers associated with M1 and M2 macrophages (Table 1). Inducible nitric oxide synthase (iNOS) , IL-6 and TNF-a were representative markers for M1 macrophages, and CD206, arginase 1 (ARG1), chitinase-like secretory lectin Ym1, resistin-like-a (Fizz1) and IL-10 were used as representative markers of M2 macrophages. Results suggested, compared with the control group, in BMDMs of COPD mice and RAW264.7 cells intervened by CSE, the mRNA expression levels of inflammatory factors and markers associated with M2 macrophages (CD206, Arg1, YM1, FizZ1, and IL-10) were increased (p<0.05, Figures 4 and 5), and the expression of iNOS was decreased (p<0.05, Figures 4 and 5). In the BMDMs from the miR-21^-/-^ model group, the mRNA expression levels of inflammatory factors and markers associated with M2 macrophages (CD206, Arg1, YM1, FizZ1, and IL-10) decreased (p<0.05, Figure 4), whereas the RNA expression level of inflammatory factors and markers associated with M1 macrophages (iNOS and TNF-a) increased (p<0.05, Figure 4B). However, there is no change in expression level of IL-6 (p>0.05, Figure 4). Adding miR-21 inhibitor to the RAW264.7 cells intervened by CSE reduced the changes of RNA expression levels of CD206, Arg1, YM1, FizZ1 and IL-10 (p<0.05, Figure 5), while the RNA expression levels of iNOS, IL-10 and IL-6 were increased (p<0.05, Figure 5).

## DISCUSSION

We discovered the miR-21 expression was increased in RAW264.7 cells intervened by CSE and gradually increased with the CSE-intervention duration. At the same time, compared with the control group, the expression of miR-21 in BDMDs cells from COPD mice was significantly increased, which was basically consistent with our previous experimental results^22^. As an important regulator, miRNAs exert a fine regulatory function on specific cellular events in the development of various organs, including the lungs. Our previous study also confirmed the differential expression of miRNAs in lung tissues of COPD rats and may have an important impact on the occurrence and development of COPD^22,29^. At the same time, our experiments suggested miR-21 is closely related to pathological changes in lung tissue. When our miR-21^- / -^ mice were modelled using the same method as the method for wild-type mice, we found that compared with wild-type mice, miR-21^- / -^ mice have significantly reduced alveolar damage, which suggests miR-21 may have an important impact on the formation of emphysema and knockout miR-21 can prevent the occurrence of emphysema as well as provide a new targeted therapy for the treatment of COPD.

Research has suggested CD68+ macrophages were increased in bronchial mucosa from COPD patients with mild/moderate stability compared with a control group^30^. Previously, the expression of CD68+ cells in the small airways was inaccurate, but now studies have shown that compared with smokers with normal lung function, the number of CD68+ macrophages in small airways increases with the severity of COPD^31^. Our experiments also verified that the protein expression of CD68 expression was increased in the COPD mouse model. It is currently unclear whether M1 or M2 macrophages are dominant in COPD, and it is possible that an intermediate phenotype exists^32^. Our previous study showed that macrophage polarization occurred in bronchoalveolar lavage fluid in COPD mice and that there was a tendency towards M2 macrophages, which were closely associated with lung function and lung tissue destruction^17^. Our experiment also confirmed that compared with the control group, the ratio of CD206/CD86 is significantly increased, which was consistent with our previous results^18^; by adopting the two continuous section-contrasted staining method we confirmed that the proportion of M2 macrophages to M1 macrophages increased in the lung tissue from COPD mouse model^18^.

MiRNAs may have an important influence and effect on the maturation of monocytes/macrophages. Our experiments verified that miR-21 inhibitors could decrease the expression of related inflammatory factors and markers associated with M2 macrophages in RAW264.7 cells with a CSE intervention. We found that M2 macrophage-related markers and factor expression were reduced in miR-21 knockout mice BDMDs cells compared with wild-type model mice, which indicated that miR-21 can mediate macrophage phenotype changes. Consistent with our observations, miR-21 is the central site for the mediation of macrophage phenotype changes from pro-inflammatory (M1) to pro-apoptotic (M2) via phagocytosis (phagocytotic apoptotic cells)^33^.

We also found that in different intervention environments, the expression of TNF-a, IL-6 and IL-10 may be different, which indicates that CSE-induced macrophage transformation may exist between M1 and M2 dynamic changes. Additionally, inflammatory cytokines and immunosuppressive factors coexist, which is a condition that results in a low but persistent inflammation and leads to COPD, and regulation of macrophage polarization can be used as a treatment for COPD patients^34^. Our experiment also confirmed this paradox, which indicates that miR-21 may exert a different effect at different stages of COPD and may lead to the coexistence of a variety of macrophage types. This requires further study to discover the role of this dynamic change in COPD. Studies have shown that pri-miR-21 may have an effect in the early stages of inflammation, while mature miR-21 has an effect on inflammation repair^35^. At an early stage of inflammation, pri-miR-21 is predominant and exhibits proinflammatory effects. In contrast, during the inflammatory repair stage, mature miR-21 exerts an anti-inflammatory effect and also causes macrophages to transform into M2 macrophages, which exhibits a low inflammation level and an immunosuppressive state and leads to the continuation of inflammation. Whether this phenomenon exists in COPD requires further study. Moreover, our previous study establishing a dynamic COPD rat model showed that miR-21 expression was a dynamic process.

### Limitations

Our experiment has some limitations. First, the mechanism of miR-21 on macrophage polarization was not studied in depth. Second, we have not studied the specific role of miR-21 in COPD, for example, epithelial-mesenchymal transition, endothelial-mesenchymal transition, and small airway remodeling etc. We thus need to further study the mechanism and the function of miR-21 and M2 macrophages in COPD.

## CONCLUSIONS

CSE can lead to macrophage transformation to the M2 phenotype and a rise in the expression of miR-21. Knockout of the miR-21 gene may reduce lung injury in COPD mouse model and could inhibit the transformation of macrophages to the M2 phenotype in COPD.

## Data Availability

The data supporting this research are available from the authors on reasonable request.
